# Molecular Basis of Interactions between the Antibiotic Nitrofurantoin and Human Serum Albumin: A Mechanism for the Rapid Drug Blood Transportation

**DOI:** 10.3390/ijms22168740

**Published:** 2021-08-14

**Authors:** Antonella Calderaro, Alessandro Maugeri, Salvatore Magazù, Giuseppina Laganà, Michele Navarra, Davide Barreca

**Affiliations:** 1 Department of Chemical, Biological, Pharmaceutical and Environmental Sciences, University of Messina, 98166 Messina, Italy; anto.calderaro@gmail.it (A.C.); amaugeri@unime.it (A.M.); giuseppina.lagana@unime.it (G.L.); davide.barreca@unime.it (D.B.); 2 Department of Mathematical and Informatics Sciences, Physical Sciences and Earth Sciences, University of Messina, 98166 Messina, Italy; salvatore.magazu@unime.it

**Keywords:** nitrofurantoin, antibiotics, human serum albumin, molecular interactions, FTIR, fluorescence

## Abstract

Nitrofurantoin is an antimicrobial agent obtained through the addition of a nitro group and a side chain containing hydantoin to a furan ring. The interactions of the antibiotic with human serum albumin (HSA) have been investigated by fluorescence, UV-VIS, Fourier transform infrared spectroscopy (FTIR) spectroscopy, and protein-ligand docking studies. The fluorescence studies indicate that the binding site of the additive involves modifications of the environment around Trp214 at the level of subdomain IIA. Fluorescence and UV-VIS spectroscopy, displacement studies, and FTIR experiments show the association mode of nitrofurantoin to HSA, suggesting that the primary binding site of the antibiotic is located in Sudlow’s site I. Molecular modeling suggests that nitrofurantoin is involved in the formation of hydrogen bonds with Trp214, Arg218, and Ser454, and is located in the hydrophobic cavity of subdomain IIA. Moreover, the curve-fitting results of the infrared Amide I’ band indicate that the binding of nitrofurantoin induces little change in the protein secondary structure. Overall, these data clarify the blood transportation process of nitrofurantoin and its rapid transfer to the kidney for its elimination, hence leading to a better understanding of its biological effects and being able to design other molecules, based on nitrofurantoin, with a higher biological potential.

## 1. Introduction

The use of antibiotics is growing every year to such an extent that there is a global effort for the development of novel therapeutics, from both a natural and synthetic origin, to combat bacterial, fungal, and viral resistance, as well as for rediscovery of the so-called “old drugs” [1]. Nitrofurantoin is a synthetic antibacterial agent widely used in the treatment of urinary tract infections [2,3,4]. It has a bacteriostatic, and, at higher concentrations, a bactericidal action on a wide range of Gram-positive and Gram-negative organisms; in particular, >90% of clinical strains of *E. coli* and *Citrobacter* spp. are sensitive [2,3,5]. After oral administration, ~40–50% of the drug is absorbed, but this value increases when nitrofurantoin is taken with food. Inside the organism, the drug is in part rapidly metabolized by the liver, and in part excreted unmodified in the urine, where it reaches a high concentration (50–250 mg L^−1^) [2,3,5,6]. Moreover, the trypanocidal activity of some synthetic analogs has been recently described [7]. The nitrofurantoin mechanism of action is complex and not well-understood, but appears to be linked to its rapid reduction by nitrofuran reductase to multiple reactive intermediates that indiscriminately attack cellular macromolecules (such us proteins and deoxyribonucleic acid) and influence metabolic energetic pathways inside the bacterial cells. On the other hand, nitrofurantoin also shows an antibacterial activity when the nitro-reductase activity is inhibited, suggesting that it may act, albeit in part, without this enzymatic reduction [8,9,10]. Some microorganisms, such as several members of the Enterobacterales order (e.g., members of the Proteae) are intrinsically resistant to nitrofurantoin, thus limiting the number of therapeutic alternatives, especially for outpatients. Nevertheless, this resistance has become a parameter utilized for their phenotypic identification/differentiation in the routine practice of microbiological laboratories [11].

Human serum albumin (HSA) is the most abundant plasma protein and is the main human carrier of several endogenous and exogenous compounds circulating in blood [12]. Therefore, the investigation of molecules based on albumin binding is of essential importance, taking into account that the bioavailability of many biologically active compounds is correlated with this affinity. The binding oftentimes induces modifications of weak interactions, which, in turn, can change the macromolecular structure and, sometimes, increase its stability [13,14,15,16,17,18,19,20]. HSA is a globular protein with three homologous domains (domains I-III) and a molecular weight of 66.5 kDa [21]. Each domain is divided into two subdomains (A and B), characterized by the presence of a α-helices structure with multiple ligand-binding sites localized in each of these subdomains [22,23,24,25]. Overall, the protein is composed of 585 amino acids, and contains 17 disulfide bridges and one free cysteine (Cys34). In this paper, the interactions between nitrofurantoin and HSA, carried out by means of a multi-spectroscopic method, are shown, providing important information such as the association and changes of the protein secondary structure. The potential site of interaction and the residues involved have been analyzed by docking studies, corroborating the data obtained experimentally.

## 2. Results

### 2.1. UV-VIS Absorption Spectra of the Nitrofurantoin−HSA Complex

**Figure 1:**
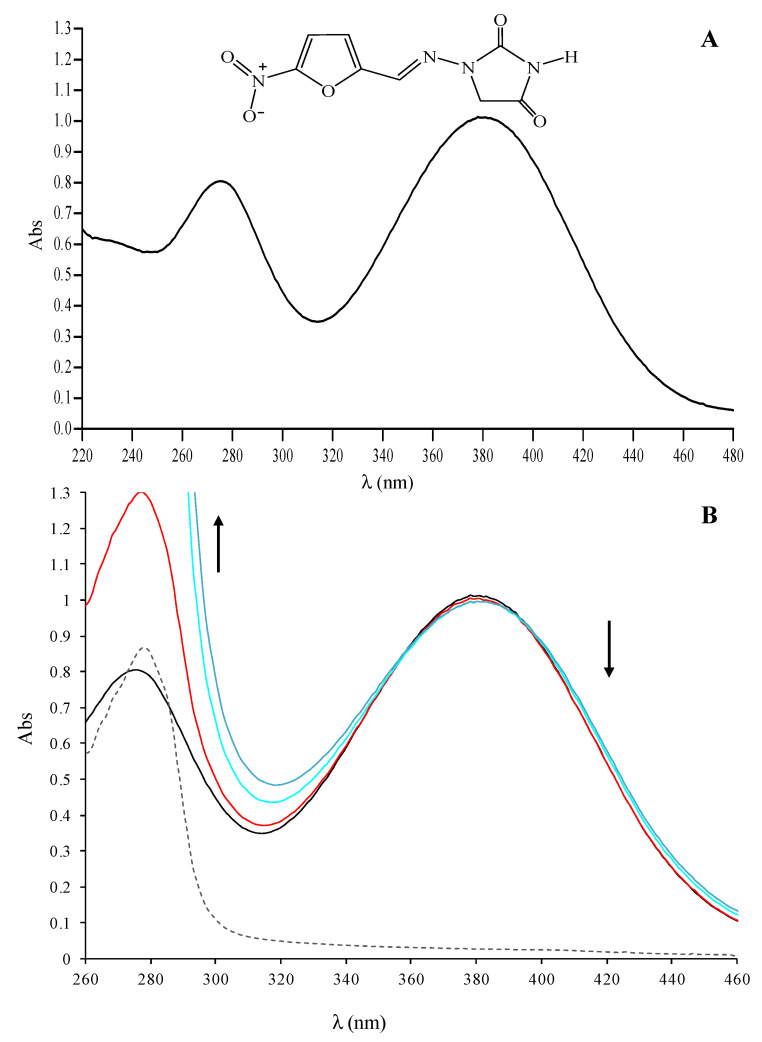
Interaction of nitrofurantoin with HSA monitored by UV-VIS spectroscopy. (**A**) UV-VIS absorption spectrum of nitrofurantoin 60 µM; (**B**) UV-VIS absorption spectra of nitrofurantoin (60 µM) in the absence (dark line) or in the presence of HSA 30 (red line), 45 (cyan line), or 60 (blue line) µM. In the graph, the spectrum of HSA 30 µM alone is also reported (dashed grey line).

UV-VIS spectroscopy supplies the first evidence of a nitrofurantoin-HSA interaction. In the range of 220–480 nm, the nitrofurantoin spectrum is characterized by three main absorption bands at 230 (shoulder), 270, and 380 nm (Figure 1A). The band at 270 nm is due to the absorption of the conjugated C=N−N unit, while those at 230 and 380 nm are due to π-π* transitions involving the nitro-substituted furan ring. The titration of nitrofurantoin (60 µM) with a solution of HSA up to 60 µM showed remarkable changes within the range 260–460 nm. Indeed, we observed a clear hypochromic shift of the maximum of absorption for the band at 380 nm, with the formation of two isosbestic points at 359 and 397 nm (Figure 1B).

This intensity decrease at 380 nm may be due to the progressive inclusion of nitrofurantoin to the HSA binding site, with the possible formation of a direct interaction between the nitro-substituted furan ring and amino acid residues able to form hydrogen bonding and apolar interactions. The titration of nitrofurantoin with HSA concentrations higher than 60 µM did not show any significant change in the absorption band, suggesting that all of the nitrofurantoin molecules are already involved in the interaction with HSA (data not shown). These results let us to suppose that the interaction between the antibiotic and the protein can be described with 1:1 stoichiometry. Less evident and of difficult interpretation are the changes in absorption at 230 and 270 nm, due to the net increase of absorbance at 280, attributable to HSA that overlaps with the two bands.

### 2.2. Fluorescence Characterization of Nitrofurantoin-HSA Complex

**Figure 2:**
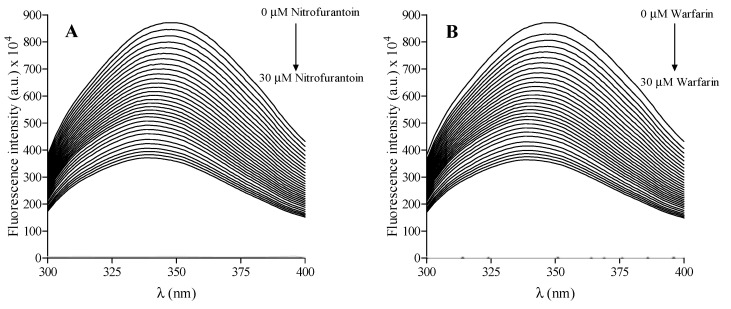
Fluorescence emission spectra of HSA in the absence or presence of increasing concentrations of nitrofurantoin (from 0 to 30 µM) (**A**) and warfarin (from 0 to 30 µM) (**B**) at 310 K.

The intrinsic fluorescence of HSA and the nitrofurantoin-HSA complex is depicted in Figure 2A, as well as that of HSA and warfarin, employed as the reference compound (Figure 2B). HSA shows a well-defined fluorescence emission with a maximum at 350 nm, due to the tryptophan residue (Trp214) present in the cavity of subdomain IIA (Sudlow’s site I). In fact, although HSA has more tyrosine than tryptophan residues, it belongs to the protein of class B, whose fluorescence emission derived from tryptophan by Forster’s resonance transfer process. The antimicrobial agent was almost non-fluorescent under the present experimental conditions. Its addition to the HSA solution gives a net decrease in the fluorescence intensity, accompanied by a shift of the wavelength emission maximum (a blue shift) in the albumin spectrum, as shown in Figure 2A. We also performed the same experiment with warfarin (a well-known compound able to bind to HSA), highlighting a similar fluorescence decease and blue shift (Figure 2B).

**Figure 3:**
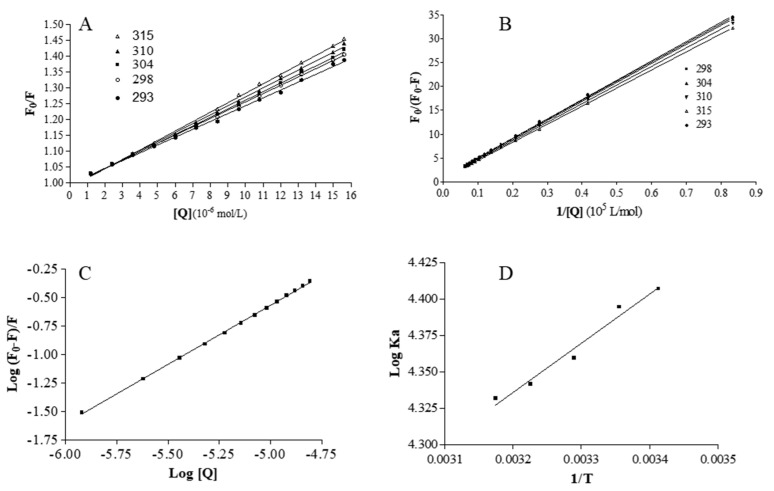
Fluorescence analysis. (**A**) The Stern−Volmer plots for the HSA-nitrofurantoin system. (**B**) Modified Stern-Volmer plots for the HSA−nitrofurantoin system. (**C**) Plots of log(F_0_-F)/F as a function of log [Q] for the binding of nitrofurantoin with HSA at the temperature of 310 K. (**D**) Van’t Hoff plot for the binding of nitrofurantoin to HSA.

Therefore, the area surrounding the tryptophan residue is highly hydrophobic. Following this, the well-known Stern-Volmer equation was employed to define the mechanism of fluorescence quenching of nitrofurantoin:

$$F_{0}/F=1+K_{\mathrm{SV}}\left[Q\right]=1+K_{q}\tau_{0}\left[Q\right]$$

where F_0_ and F are the fluorescence intensities of HSA in the absence and in the presence of nitrofurantoin, K_q_ is the quenching rate constant, K_SV_ is the Stern−Volmer dynamic quenching constant, τ_0_ is the average lifetime of the fluorophore in the absence of quenchers, and [Q] is the concentration of the quencher. The Figure 3A depicts the curves of F_0_/F versus [Q] at different temperatures. The K_SV_ values obtained at 293, 298, 304, 310, and 315 K are 2.48 (±0.32) × 10^4^, 2.63 (±0.22) × 10^4^, 2.7 (±0.18) × 10^4^, 2.86 (±0.30) × 10^4^, and 2.98 (±0.27) × 10^4^ M^−1^, respectively. Nitrofurantoin is soluble in a buffer solution and this may accelerate its diffusion rate and hence its collision with fluorophore.

The fluorescence data were further examined using the modified Stern−Volmer equation:

$$\frac{F_{0}}{F_{0}-F}=\frac{1}{f_{a}K_{a}\left[Q\right]}+\frac{1}{f_{a}}$$

where K_a_ is the modified Stern−Volmer association constant for the accessible fluorophores, and f_a_ is the fraction of accessible fluorescence. The linear regression analysis of F_0_/(F_0_-F) versus 1/[Q] is shown in Figure 3B. The obtained K_a_ values are 2.55 × 10^4^, 2.48 × 10^4^, 2.28 × 10^4^, 2.19 × 10^4^, and 2.15 × 10^4^ L mol^−1^ at 293, 298, 304, 310, and 315 K, respectively. This shows that the binding constant is moderate and the effect of temperature is not significant. The analysis of the plots showed that, at the tested concentrations, there is a good linear relationship, indicating that the quenching mechanism is driven by the formation of a complex.

### 2.3. Analysis of Binding Equilibrium

The binding ability of a compound to HSA is very useful for evaluating its biological and, eventual, therapeutic potential, because it can also influence its stability and toxicity, as well as the rapidity of the renal excretion. The independent bind of small molecules to a set of equivalent sites on a macromolecule can be described by analyzing the equilibrium between free and bound molecules using the following equation:

$$\mathrm{Log}\left(F_{0}-F\right)/F=\mathrm{LogK}+\mathrm{nlog}\left[Q\right]$$

where K is the observed binding constant to a site and *n* is the number of binding sites per HSA. In Figure 3C, the linear plot of log (F_0_-F)/F as a function of log[Q] at 310 K is shown. The values of *n* are approximately equal to 1, which demonstrates that there is a single class-binding site for nitrofurantoin in the proximity of the tryptophan residue.

The binding strength of a compound to HSA is one of the main elements in its availability to diffuse in the organism and, through the circulation system, to reach its target organ or to be eliminated by the organism [26]. The binding of a ligands to HSA and proteins, in general, is often reversible with moderate affinities (binding constants in the range of 1–15 × 10^4^ L/mol) [27]. The binding constant (K) obtained at the physiological temperature value (310 K) is 4.10 ± 0.02 × 10^4^ L/mol, highlighting that the binding between nitrofurantoin and HSA is moderate in strength, and the formation of the complex is reversible. In this way, the antibiotic can be stored and carried around the body by HSA. The moderate value of K indicates that the drug does not remain so much time linked to HSA because other molecules with higher affinity compete with nitrofurantoin for the binding, and this decreases its availability in the circulating system and probably is responsible also of its rapid elimination.

### 2.4. Thermodynamics and Acting Forces

The binding of molecules to macromolecules involves mainly non-covalent interactions (such as hydrogen bonds, van der Waals forces, hydrophobic, and electrostatic interactions). These types of forces can be analyzed by determining the thermodynamic parameters of the binding reaction [13,28,29,30,31,32,33,34,35]. Therefore, the thermodynamic parameters dependent on temperature were calculated from the van’t Hoff plot to characterize the forces acting between nitrofurantoin and HSA. When the temperature change is not very large, the enthalpy change (ΔH) of a system can be regarded as a constant. Under these conditions, both the enthalpy (ΔH) and entropy (ΔS) changes can be evaluated from the van’t Hoff equation:

$${\mathrm{LnK}}_{a}=-\frac{\Delta H}{\mathrm{RT}}+\frac{\Delta S}{R}$$

where R is the gas constant. The enthalpy change (ΔH) is calculated from the slope of the van’t Hoff plot (Figure 3D), while the entropy change (ΔS) is calculated from the intercept. The free energy change (ΔG) is then estimated from the following equation:

ΔG=ΔH−TΔS

The van’t Hoff plot for the evaluation of the thermodynamic parameters due to the nitrofurantoin-HSA interaction is depicted in Figure 3D. The ΔH, ΔS, and ΔG values are depicted in Table 1.

The positive ΔS value is evidence for the formation of hydrophobic interactions, while the negative value of ΔH suggests that the binding process is predominately enthalpy driven and is probably due to hydrogen binding interactions. The negative ΔG values, accompanied by positive entropy change (ΔS), are indicative of a spontaneous process during the binding of nitrofurantoin to HSA. Moreover, the free energy change in the binding is a direct consequence of the strength of the interaction (such as protein molecules). Therefore, both hydrophobic interactions and hydrogen bonds play a major role in the binding of an antibiotic agent to HSA according to above mentioned data.

### 2.5. Fluorescence Displacement Binding Experiments

**Figure 4:**
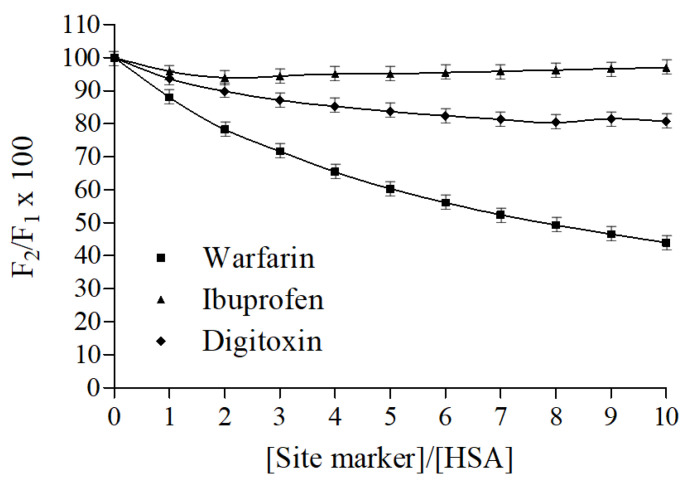
Effect of site-specific ligands on the fluorescence of the HSA-nitrofurantoin complex. F_1_ and F_2_ are the fluorescence of HSA−nitrofurantoin in the absence and presence of the site markers, respectively. Data represent mean ± SD (*N* = 3).

Warfarin, ibuprofen, and digitoxin, namely markers of HSA binding sites I, II, and III, respectively, were employed to carry out displacement binding experiments and to gather further information on the nitrofurantoin binding site. The fluorescence emission spectra of nitrofurantoin-HSA complex spectra were recorded both in the absence and presence of increasing concentrations of warfarin, ibuprofen, and digitoxin. As displayed in Figure 4, The fluorescence of HSA-nitrofurantoin rapidly decreased after warfarin addition, whereas it remained almost unvaried regardless of the presence of both ibuprofen and digitoxin. As warfarin competes with nitrofurantoin for the same HSA binding site, this supports the fact that the latter probably binds to the hydrophobic pocket situated in subdomain IIA (Sudlow’s site I). These results are in line with those above reported, and suggest that the residue Trp214 should be close or inside the binding site of nitrofurantoin.

### 2.6. Conformation Investigation by Fourier Transform Infrared Spectroscopy (FTIR) Spectroscopy

**Figure 5:**
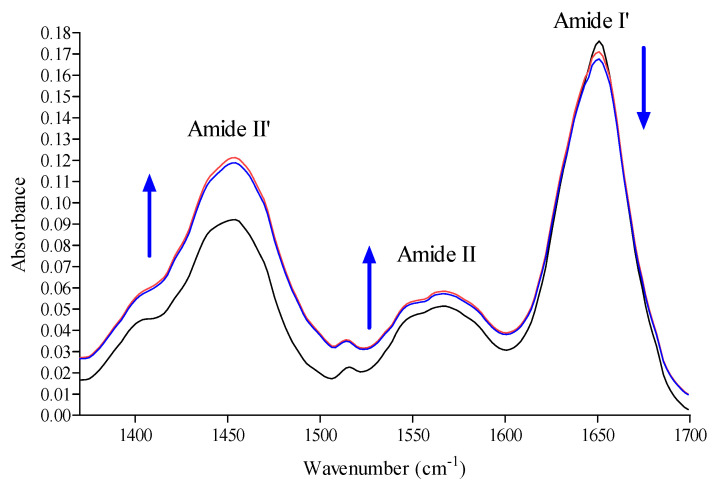
FTIR absorption spectra of HSA at 310 K in absence (black line) or presence of nitrofurantoin (red line) and warfarin (blue line). The arrows indicate the changes in the main amide bands after interaction with nitrofurantoin or warfarin.

In order to investigate the modification of the HSA secondary structure after binding to nitrofurantoin, as well as its mechanisms of interaction to the protein, we exploited FTIR spectroscopy [36,37,38,39,40,41]. The HSA secondary structure is characterized by 67% α-helix, 10% turn, and 23% extended chains. As shown in Figure 5, HSA exhibited a strong Amide I′ band centered at around 1652 cm^−1^, corresponding mainly to the α-helix. In addition, the interaction between nitrofurantoin and HSA brought about a change, as well as a slight shift in band intensity, at both 1652 cm^−1^ and 1680 cm^−1^ of the FTIR spectra, thus suggesting a rapid variation in the association and dissociation of nitrofurantoin. This was further reinforced by the observed increase of absorbance values of both amide II’ and amide II bands.

The absorption of these two bands arises from the amide bonds that link the amino acids. The absorption is primarily due to bending vibrations of the N−H bond and, because they are involved in hydrogen bonding occurring among the different elements of a secondary structure (as well as in H–D exchange), they are sensitive to the changes in secondary structure and to the interaction with the surrounding environment of the protein.

### 2.7. Molecular Modeling Study

**Figure 6:**
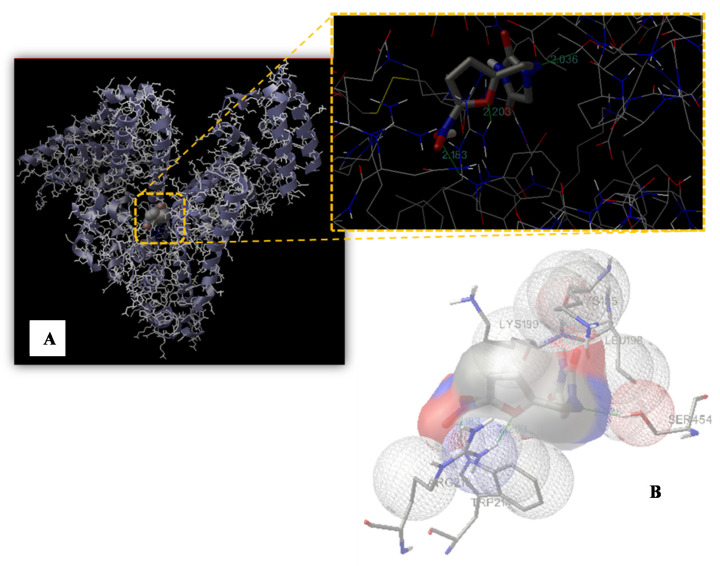
Binding site of nitrofurantoin to HSA. (**A**) The HSA structure is rendered with ribbons and lines, while nitrofurantoin is rendered as space fill. The inset shows a magnification of the binding site with nitrofurantoin represented using a stick model. The hydrogen bonds between the ligand and the protein are shown in green and the distance is expressed in Å. (**B**) Representation of the amino acid residues in the binding site with their van der Waals radii.

Our computational modeling study was performed on a crystal structure of HSA taken from the Protein Data Bank (entry PDB code 1GNI) in order to identify the possible binding site of nitrofurantoin. Based on the displace experimental results and to further define the binding site, the molecule docking simulation box was set on Sudlow’s site I. The best energy ranked result (−5.43 kcal/mol) showed that nitrofurantoin may be situated within subdomain IIA, formed by six helices, which is consistent with the supposition on the basis of our experimental results. The nitro group and the oxygen of the furan ring established a hydrogen bond with Trp214 and Arg218, respectively, and the C=N−N unit of hydantoin established a further one with Ser454 (Figure 6). The nitrofurantoin ring is located within the binding pocket with the nitro group and the furan ring, which protrudes from it. The hydrogen bond formed with Arg218 and Ser454, as well as the one with Trp214, indicates that the interaction between nitrofurantoin and HSA is not exclusively hydrophobic in nature, but involves ionic and polar interactions. Although nitrofurantoin and warfarin bind to the Sudlow’s site I within in the subdomain IIA, they do not share the same binding region. The warfarin binding region is located inside the hydrophobic pocket of the IIA subdomain and, in its vicinity, there are four positively charged residues (Lys199, Arg222, His242, and Arg257) and three apolar residues (Tyr150, Leu238, and Leu260). Warfarin also forms three hydrogen bonds with Tyr150, Arg222, and His242 [42,43,44].

## 3. Discussion

The identification and characterization of novel antibiotics for clinical utilization is one of the greatest challenges of current basic experimentation. Indeed, discoveries in this field have always represented a remarkable achievement in the history of medicine. Moreover, the development and diffusion of bacterial antibiotic-resistance, due to the modification of different proteins and hence cellular processes (i.e., enzymatic degradation, molecular target alteration, decrement of drug uptake, overexpression of specific efflux pump proteins, etc.), is a major public health issue. Therefore, researchers are continuously trying to develop new agents of both a natural and synthetic origin that are able to overcome this resistance and fight the major issue bacterial infections still represent [45,46,47]. This may be achieved by enhancing the basic knowledge of biological processes and, sometimes, by repurposing “old” drugs in order to overcome the most difficult periods. Nitrofurantoin is an antibiotic widely used in medicine, but the analysis of the literature allowed us to notice a lack of information regarding its blood transportation and its interaction/binding with either serum/plasma proteins. The interaction and eventually binding with “carrier proteins” (such as HSA) is fundamental for the evaluation of the biological potentials of drugs, in general, and antibiotics, in particular, as it is actually one of the steps during antibiotic drug development [48]. In our study, the obtained spectroscopic results clearly highlighted the potentiality of HSA to bind nitrofurantoin and to drive it around the circulating system. The binding involved the formation of weak interactions, in particular between the nitro-substituted furan ring and amino acid residues able to form hydrogen bonds and apolar interactions at the level of the hydrophobic pocket located in subdomain IIA (Sudlow’s site I), as shown by UV-VIS and fluorescence spectroscopy. Moreover, the data obtained for K_SV_ in the Stern-Volmer dynamic quenching equation and the evaluation of the other thermodynamic and kinetic parameters indicate that there is the formation of a binary complex in a single site in the neighboring of a tryptophan residue. Both hydrophobic interactions and hydrogen bonds are acknowledged to have a crucial role in the binding of nitrofurantoin to HSA, as in the case of other antibiotics [49]. The binding clearly affects the secondary structure of HSA, given the decrease in the percentage of the α-helix structure, as well as the change in absorbance values of both amides I and II after FTIR analysis. The vibration modes of these two bands are affected by both the conformation and environment of the amide group. This increase is given by the H–D exchange, which involves hydrogen within the core, thus justifying the formation of transitory protein conformational states during the HSA-nitrofurantoin complex formation. The increment of the amide II band may be a characteristic of hydrogen bonding formation during this process. Indeed, this affects mostly N-H bending vibration, which contributes exclusively to amide II vibration, which is more sensitive to H-D exchange. Starting from these observations and following molecular modelling data, we have shown that weak interactions play an important role in stabilizing the complex HSA−nitrofurantoin, and shed light on the results obtained by UV-VIS and fluorescence spectroscopy. Moreover, the hydrogen bond at the level of the C=N−N unit of nitrofurantoin with Ser454 contributes to its orientation and spatial arrangement inside the Sudlow’s site I and provides experimental evidence to explain the fluorescence quenching of HSA emission in the presence of the antibiotic and the decrease in the absorbance of the band at 380 nm. Both changes are probably attributable to the formation of the hydrogen bond of the nitro-substituted furan ring with Trp214 and Arg218, influencing not only the quenching of HSA fluorescence, but also the π-π* transitions observed by UV-VIS spectroscopy. As reported in the results, both nitrofurantoin and warfarin bind to the same binding site and induce a change in the secondary structure content of the protein, although both the region of interaction and the mode of binding are different. This is due to the flexibility of this site and the possibility to bind different molecules (such as large heterocyclic and negatively charged compounds) in a specific zone, characterized by the presence of selected amino acid clusters.

## 4. Materials and Methods

### 4.1. Materials

Both HSA and nitrofurantoin were purchased from MERCH (Darmstadt, Germany, Europe). We employed no fat-free HSA so as to resemble a much closer situation closer to what occurs in vivo. Double distilled water was employed to prepare all of the solutions.

### 4.2. UV-VIS Spectra

UV-VIS spectroscopy was employed to study the interaction between HSA and nitrofurantoin, adding increasing amounts of the former (up to 90 µM) to 60 µM of the latter in a 20 mM phosphate buffer with pH 7.4. The absorption spectra were recorded from 220 to 480 nm through a spectrophotometer with a quartz cuvette. The spectra of each buffer solution were subtracted from the sample ones.

### 4.3. FTIR Spectra

Lyophilized HSA was dissolved in D_2_O at 298 K for 1 day and was lyophilized again. This procedure was carried out twice for each sample. The FTIR spectra (1350 cm^−1^and 1750 cm^−1^) were measured through a Bruker Vertex 80 spectrometer under a vacuum, after dissolving the samples in 20 mM phosphate buffer with pH 7.4 in D_2_O. The pD value of the HSA solution was measured and corrected according to pD = pH + 0.4 for deuterium isotope effects. A pair of CaF_2_ windows divided by a 25 μm Teflon spacers were employed to place the protein solution (30 mg/mL), with or without nitrofurantoin or warfarin. Sixty-four interferograms with a spectral resolution of 4 cm^−1^ were collected for each sample. The IR spectra of the buffer with or without nitrofurantoin or warfarin were subtracted from the spectra of the corresponding sample.

### 4.4. Data Analysis of FTIR Spectra

The HSA spectra with or without nitrofurantoin or warfarin were smoothed by employing the Loess algorithm, and the deconvolved spectra were fitted with Gaussian band profiles. From these spectra, the initial values for the peak heights and widths were assessed. For the final fits, the positions, heights, and widths of each band were varied simultaneously. The curve fitting process was calculated through Seasolve PeakFit v4.12 software.

### 4.5. Fluorescence Spectra

The fluorescence spectra were recorded using a FluoroMax-4 spectrofluorometer by Horiba Jobin-Yvon, equipped with a pulsed xenon lamp and an F-3006 Autotitration Injector with two Hamilton Syringes (mods. Gastight 1725 and 1001 TLLX, with a 250 µL and 1.0 mL capacity, respectively). The resolutions of the wavelength selectors and titrant additions were 0.3 nm and 0.25 µL, respectively. The instrument was also equipped with a Peltier Sample Cooler (mod. F-3004) controlled by a Peltier Thermoelectric Temperature Controller model LFI-3751 (5 A–40 W). The whole system was controlled by the FluorEssence 2.1 software by Horiba Jobin-Yvon. The titrations were performed directly in a Hellma type 101-OS precision cell (Light Path 10 mm), where a magnetic stirrer and the anti-diffusion burette tip were placed in a position that would not interfere with the light beam. The automatic data acquisition (fluorescence intensity vs. λ(nm) for each titrant addition) was performed using the same FluorEssence 2.1 software. The excitation and emission bandwidths were both 5 nm. The protein samples were excited at 280 and 295 nm in order to characterize the possible different behavior of tryptophan and/or tyrosine residues. It was observed that both spectra were similar. The rest of the experiment was acquired by excitation at 280 nm and the emission spectra were recorded in the range of 300–400 nm. The potential interaction between nitrofurantoin and HSA was performed by fluorimetric titration. A 2.0 mL solution containing 1.5 × 10^−5^ mol/L HSA in 20 mM sodium phosphate buffer (pH 7.4) was titrated by a successive additions of nitrofurantoin stock solution (1.0 × 10^−3^ mol/L) to give a final concentration ranging from 0 to 3.0 × 10^−5^ mol/L. The fluorescence spectra were recorded at 293, 298, 304, 310, and 315 K in the wavelength range of 300−400 nm with an excitation wavelength at 280 nm.

The displacement studies were carried out utilizing different site markers (warfarin, ibuprofen, and digitoxin for sites I, II, and III, respectively). A solution of HSA (1.5 × 10^−5^ mol/L) in a 20 mM sodium phosphate buffer (pH 7.4) containing nitrofurantoin at the same final concentration (1.5 × 10^−5^ mol/L) was titrated by successive additions of site markers solution (1.0 × 10^−3^ mol/L) to obtain overall site markers concentrations ranging from 0 to 9 × 10^−5^ mol/L. Fluorescence spectra were recorded at 310 K in the range of 300-400 nm with an excitation wavelength at 280 nm. The fluorescence of the ternary mixture as a percentage of the initial fluorescence was determined according to the method of Sudlow et al. [50]:

$$\frac{F_{2}}{F_{1}}\times 100$$

where F_1_ and F_2_ are the fluorescence of HSA−nitrofurantoin in the absence and in the presence of the site markers, respectively.

### 4.6. Molecular Modeling Study

Docking calculations were carried out using AutoDock 4.2, and were graphically rendered by PyMOL 2.5 [51,52]. The MMFF94 force field was used for energy minimization of the ligand molecule (TC) using DockingServer. Gasteiger partial charges were added to the ligand atoms. Nonpolar hydrogen atoms were merged and rotatable bonds were defined. Docking calculations were carried out on an HSA protein model (PDB code 1GNI) and on nitrofurantoin, whose coordinates were taken from the ZINC database (entry code 7997568). Essential hydrogen atoms, Kollman united atom type charges, and solvation parameters were added with the aid of AutoDock tools. Affinity (grid) maps of 50 × 32 × 40 Å grid points and 0.375 Å spacing were generated using the Autogrid program. The AutoDock parameter set and distance-dependent dielectric functions were used in the calculation of the van der Waals and the electrostatic terms, respectively. Docking simulations were performed using the Lamarckian genetic algorithm (LGA). Initial positions, orientations, and torsions of the ligand molecules were set randomly. All of the rotatable torsions were released during docking. Each run of the docking experiment was set to terminate after a maximum of 250,000 energy evaluations. The population size was set to 150. The conformer with the lowest binding free energy was analyzed and used for further determinations.

## 5. Conclusions

The present experimental work describes, for the first time, the binding of nitrofurantoin to HSA. Our results may describe one of the mechanisms underlying the blood transportation of this compound through our body up to the kidney for its elimination. The binding reaction is spontaneous and involves hydrogen bond and hydrophobic interaction, as highlighted by the changes in the thermodynamic parameters. The interactions were confirmed through alterations in the UV−VIS absorption spectra of nitrofurantoin and albumin and the quenching of the intrinsic fluorescence of the protein, probably due to the hydrogen bond and apolar interactions with the nitro-substituted furan ring. In addition, the nitrofurantoin binding site is located in the hydrophobic pocket of subdomain IIA according to the site competitive study. Altogether, our results help to achieve a better understanding of the pharmacokinetic of nitrofurantoin, in order to design analogous molecules with a greater biological potential.

## Figures and Tables

**Table 1:** Analysis of the thermodynamic parameters.

ΔH.	ΔS	ΔG				
(kJ mol^−1^)	(J mol^−1^K^−1^)	(kJ mol^−1^)				
		293	298	304	310	315
−3.93 ± 0.26	27.05 ± 0.85	−11.32 ± 1.25	−11.45 ± 1.11	−11.62 ± 1.13	−11.78 ± 1.41	−11.91 ± 1.72

## Data Availability

Not applicable.
